# Analyzing and Prioritizing the Dimensions of Patient Safety Culture in Emergency Wards Using the TOPSIS Technique

**DOI:** 10.5539/gjhs.v7n4p143

**Published:** 2014-12-31

**Authors:** Sogand Tourani, Mahdi Hassani, Ali Ayoubian, Mansooreh Habibi, Rouhollah Zaboli

**Affiliations:** 1 Health Management and Economics Research Center, Iran University of Medical sciences, Tehran, Iran; 2 Police Medicine Research Center, Tehran, Iran; 3 Health Research Center, Baqiyatallah University of Medical Sciences, Tehran, Iran; 4 Hospital Management Research Center, Iran University of Medical Sciences, Tehran, Iran; 5 Health Management Research Center, Baqiyatallah University of Medical Sciences, Tehran, Iran

**Keywords:** culture, patient safety, hospital departments, emergency service, hospital

## Abstract

**Background and Aim:**

Doubtlessly, permanent development in patient care services is not feasible without paying attention to the culture of safety by health and treatment institutes. The present study is an attempt to analyze the cultural aspects of patient safety in the emergency wards of hospitals affiliated with the Tehran Medical Science University. The viewpoint of the nurses and hospital officials and their priorities were studied. For prioritizing the results of this study the TOPSIS technique was chosen.

**Methods:**

The study was conducted as an analytical-descriptive and cross-sectional one. It was carried out in two parts: at first the cultural aspects of the patients were measured using a questionnaire for a six months period in 2011 in emergency wards of the hospitals under study. The study population was constituted of physicians and nurses of the emergency wards. The sample group (n=270) was selected through a cluster sampling and its size was determined by using the sample size formula. For data gathering, the standard questionnaire of Hospital Survey on Patient Safety Culture (HSOPSC) was used. The data were analyzed in SPSS. The aspects of the safety culture were prioritized using the TOPSIS model. The criteria were ranked by using the MATLAB software.

**Results:**

There was a significant relationship among the aspects of performance, teamwork, feedback, mistake relationships, and the support of the managers (P ≤ 0.05). The total point of the patient safety culture in the majority of the hospitals were at a mean level of 3. The maximum score was 5. The maximum and minimum mean points were obtained by the Hasheminejad and Sina hospitals respectively. The results of the multivariate decision-making analysis indicated that human, managerial, organizational, and environmental factors were at the top of priorities in a descending order. The factors were extremely effective in the improvement of safety in hospitals.

**Conclusion:**

Human factors were the most effective and important factors in the improvement of safety in emergency wards. Therefore, there is a need to pay more attention to such factors in safety improvement programming. Training, cultural works, preparation of organizational environments, and motivating environmental factors were of the main measures that must be taken into account by the managers.

## 1. Background

Many patients face injuries and deal with many other problems caused by medical errors, infections caused by medical interventions, errors during surgery and so on (Bishop & Boyle, 2014). These are happening while the final goal of health and treatment services is to keep the patients safe and take care of them (Chang et al., 2012). One of the clearest human rights is the right of being safe from the possible hazards throughout provision of health services (Aboul-Fotouh, 2012). However, the shocking fact is that medical errors are one of the key challenges of health systems in many countries (Goodman, 2003). The idea that health systems in many countries are not safe enough and needto improvement has been pushed forward in the last two decades. On the other hand, safety improvements and developments regarding the services received by the patients have helped health organizations and clinics to spot the threats and find solutions to deal with the problems. Obviously, a permanent and immediate improvement is not possible without the promotion of safety cultures on every aspect of health services (Lange et al., 2011).

Safety is one of the main issues of clinical services. The Associations of Physicians recommended improvements of the patient safety to health institutes in 2000. Improvements regarding the issues pertinent to organizational culture were one of the proposed ways to this end (Ilan et al., 2005). As indicated by the evidences, the problems caused by lack of patient safety are mainly rooted in organizational systems approach while little room is left for individual’s error (Keady et al., 2008). As suggested by studies, implementation of a systematic approach to medical error needs changes in the system, organizational safety culture, accident reporting system, and analyzing the results. This ensures the improvement of the safety of patients and the effectiveness of clinical services, while it is also helpful in the improvement of the managements’ efficiency and the reduction of costs. The implementation of a systemic approach to error preventions leads to shorter hospitalization periods, medicine use, and medical issues caused by side effects, reduction of costs, and prolonged hospitalization (Dalton et al., 2008; Shah et al., 2012). It is estimated that, 239000 patients died under Medicare between 2004 and 2006 in the USA merely due to medical errors. Furthermore, 24000 died because of medical errors in Canada (Sharma et al., 2011; Woolhandler et al., 2003). In addition to death and inabilities, medical errors impose considerable costs to the health sector. As indicated by reports, medical errors cost $8.8 billion in the USA Medicare program between 2004 and 2006 (Sharma et al., 2011).

A great deal of work is done towards the improvement of patients’ safetymainly done after incidents. However, higher potentials can be explored in spotting and removing threats before they cause any trouble (Reson, 2000). To ensure success in this regard, the patients’ safety must be a priority and all the measures taken by the organization must be directed in this line (Pronovost et al., 2003).

The traditional culture of punishing the employees for the errors hinders the possibility of learning from the errors; an issue that is a heated debate. The importance of cultural work in health organizations grows day by day (Hemman, 2003). In spite of the great deal of emphasis regarding patients’ safety, few organizations have managed to reevaluate cultural support for the patients’ safety. Helingz et al. concluded that many aspects of safety culture are in poor conditions (Hellings et al., 2007). A report on safety culture in 622 hospitals located in the USA by the Health Research and Quality Agency (2009) indicated teamwork attitudes in the ward as the main aspect of safety culture (79% positive response), while non-punishing reactions to errors (44% positive responses) was named as the weakest aspect of safety culture (Patel, & Wu, 2014). A study in Turkey (2009) showed that the teamwork index of each ward (76%) gained the highest positive response after general perceptions of safety (59%). On the other hand, the frequency of reporting incidents (12%) and non-punishing responses to errors (18%) obtained the lowest points (Bodur & Filiz, 2009). The common program reported by many studies indicates that the leadership of the organizations believes that safety culture is a bedrock for all future improvements in patient safety in the institutes; and thus, these organizations have initiated safety culture development programs (Dalton et al., 2008).

Safety is critical in the provision of emergency services as it deals to life of the human beings or influences lives of others. Emergency wards of hospitals are the first place which patients receive medical attention. The ward is the entrance of the hospital service provision systems. Given the specific nature of the services, complexity, frequency, and urgency the performance of the emergency ward has a critical role in reducing mortality rates, protection of publish health, and the improvement of the public satisfaction with medical services. Due to physical weaknesses, inability to resist, and lack of enough physical activities, hospitalized patients need extra support and attentions (Trzeciak & Rivers, 2003).

As indicated by studies, the better the safety atmospherethe fewer the problems the patients would have regarding the patients’ safety (Zohar et al., 2007). Therefore, improvement of the patient safety culture and transfer from the zero-error culture to the culture that motivates error reporting even when no harm is done to the patients are vital in health providing organizations (Wilson, 2009). To put it in another way, identifying safety culture in the hospitals and emergency wards in particular is essential to improve the patients’ safety and consequently improves the quality of services, medical care, and the correction of probable problems.

## 2. Objective

The present study is a comparative analysis of the aspects of the patients’ safety in emergency wards of the hospitals affiliated with the Tehran Medical Science University from the viewpoint of nurses and physicians of emergency wards. In addition, these aspects were prioritized using the TOPSIS technique.

## 3. Methods

This cross-sectional study was carried out as a descriptive-analytical research. The main point of the cultural aspects of the patients’ safety in emergency wards was obtained based on questionnaires. The questionnaires were administered in a six months period in 2011 in the emergency wards of ShahidHasheminejad, Firouzgar, HazratFatemeh, HazratAliasghar, HazratRasoulakram, Imam Khomeini, Shariate, Razi, Sina, Farabi, and Children Medican Centers. The study population was comprised by all the physicians and nurses working in emergency wards. Totally, 270 participants were selected through cluster samplings.

For data gathering, the standard questionnaire HSOPSC was used. The questionnaire was designed by the Agency of Health Research and Quality (AHRQ) in 2004 (Zohar et al., 2007). The questionnaire is comprised of 42 statements that focus on 12 different aspects of the patients’ safety (frequency of incident reporting, general perception of the personnel of the patients’ safety, measures taken by supervisors to ensure the patients’ safety, organizational learning, teamwork attitude in the ward, communication lines, error feedback, non-punishing responses to errors, the employees’ issues, managements’ support of the patients’ safety, teamwork attitude among wards, and information sharing). This questionnaire was based on the Likert’s five-point scale (1=completely disagree - 5=completely agree). Data were analyzed by using the SPSS software. The normal distribution of the data was tested before the inferential statistical test (one-way variance analysis).

To prioritize the cultural aspects of safety, the TOPSIS technique was used. Among the variety of techniques for making decisions with multi variables, TOPSIS has considerable merits. The technique is a multivariate decision-making method in which M items are ranked based on N criteria. The technique was first introduced by Howang, Lee, and Lai (Yoon, & Hwang, 1998). The adopted alternatives must be as close as possible to the best alternative and as far as possible to the worst alternative. TOPSIS is featured with six steps:

1) Create an evaluation matrix consisting of normalizations;
2) Normalized matrix is weighted by weighting the criteria;
3) Determine best and worst alternatives;
4) Calculate the distance of each alternative to positive and negative alternatives
5) Calculate relative similarities of an alternative to the best alternative and ranking the alternatives (Jahanshahloo et al., 2006; Tzeng & Huang, 2011).

To prioritize, a group of experts was formed.

Based on analyzing the mean point of 12 factors in the patient safety culture, the experts categorized the items into three categories of human factors, managerial factors, organizational and environmental factors. The criteria of decision-making were determined for creating the decision-making matrix. The decision making criteria (C1, C2, C3, C4) were the effect on reducing mortality, effectiveness and efficiency, benefits cost, acceptance by employees and ease of implementation. The experts (D1, D2 D3, D4) used very poor (VP), poor (P) moderate (M), good (G), very good (VG) to weight the criteria. The criteria were selected and weighted by the experts. Afterwards, the matrix was normalized and calculations were done (diagonal multiplication) and the ideal decision-making matrix (negative and positive) was determined and eventually the distances to ideal alternatives were obtained and prioritization was carried out. The criteria were ranked by using the MATLAB software.

## 4. Results

The mean point of the aspects of the patients’ safety culture in the studied emergency wards showed significant differences between the aspects of performance of supervisors to improve the patients’ safety, team works in the ward, feedback and communicating errors and supportive attitudes of the management (P≤0.05). The findings showed no significant difference among the hospitals regarding the point of the patients’ safety culture (p>0.05). The total point of the patients’ safety culture in the majority of the hospitals was at the mean level (i.e. 3). In addition, the Hasheminejad and Sina Hospitals had the highest and lowest points (Table 1).

**Table 1:** Mean point of different aspects of the patient safety culture in the emergency wards

Aspects of the patient safety culture	ShahidHasheminejad	Firouzgar	HazratRasoulakram	HazratFatemeh	Farabi	Razi	Sina	Children Medical Center	Women Hospital	Shariati	Imam Khomeini	HazratAliasghar	P-Value
	μ±δ	μ±δ	μ±δ	μ±δ	μ±δ	μ±δ	μ±δ	μ±δ	μ±δ	μ±δ	μ±δ	μ±δ	
Reporting errors pertinent to patient safety	0.87±3.22	0.84±2.88	0.79±3.1	0.72±3.03	1.00±3.48	1.26±3.86	0.69±3.19	0.59±3.54	0.74±3.37	0.65±2.98	0.69±3.22	0.58±2.75	0.13

Performance of the supervisors to improve patient safety	0.86±3.6	0.64±3.08	0.58±2.94	0.56±3.19	0.78±3.16	0.54±3.39	1.12±2.55	0.71±3.51	0.69±3.56	0.73±2.77	0.63±3.16	0.69±3.47	0

Teamwork attitude regarding patient safety in different wards	0.62±4.03	0.70±3.35	0.84±3.3	0.60±3.42	0.92±3.48	0.43±4.11	0.63±2.7	0.75±3.75	0.87±3.73	0.50±2.96	0.54±3.77	0.36±3.53	0

Training and permanent development regarding patient safety	0.64±3.8	0.53±3.63	0.71±3.28	0.68±3.43	0.79±3.15	0.63±3.86	1.07±3.29	0.66±3.67	0.89±3.59	0.74±3.31	0.66±3.6	0.73±3.88	0.112

Responses to reports of errors	0.87±3.78	0.86±3.42	0.61±3.3	0.77±2.97	0.70±3.35	0.62±2.71	1.15±2.76	0.95±2.98	1.03±3.34	0.74±2.6	0.90±3.15	0.84±3.33	0.009

Management support of patient safety measures	0.53±3.8	0.59±3.75	0.65±3.1	0.82±3.5	0.41±3.54	0.72±3.48	1.30±2.21	0.85±3.49	0.76±3.47	0.91±2.64	0.80±3.3	0.98±3.33	0

Emergency ward staff work load	0.66±2.84	0.56±2.3	0.68±2.18	0.67±2.71	0.51±2.46	0.44±2.79	0.49±1.79	0.65±2.35	0.59±2.32	0.82±2.05	0.82±2.03	0.73±2.66	0

Quality of the patient’s information shared by the wards	0.58±3.32	0.72±3.45	0.71±3.05	0.71±3.23	0.86±2.6	0.61±3.64	1.32±3.04	0.90±3.34	0.63±3.75	0.92±2.48	0.67±2.67	1.12±3.25	0

Open and transparent communications regarding the incidents	0.77±3.2	0.71±3.08	0.61±3.19	0.72±3.04	0.58±3.58	0.42±2.86	0.76±2.81	0.85±2.96	1.08±3.06	1.02±2.69	0.80±3.21	0.66±3.92	0.013

Non-punishing responses to errors	0.75±2.39	0.75±2.19	0.58±2.31	0.59±2.14	0.54±2.05	1.03±2.48	0.70±1.91	0.45±2.26	0.80±1.96	0.86±2.07	0.79±2.38	0.49±2.04	0.404

Teamwork among different wards	0.66±3.4	0.75±2.94	0.64±2.94	0.74±3.21	0.82±3.28	0.99±3.57	0.83±3.07	0.48±3.23	0.75±3.24	0.85±2.88	0.65±2.98	1.18±3.03	0.359

General perception of the patient safety among emergency ward staff	0.72±3.49	0.84±3.16	0.62±3.25	0.73±3.15	0.72±3.3	0.74±3.58	1.07±2.71	0.61±3.69	0.73±3.55	0.74±2.39	0.75±2.98	0.50±3.66	0

General status of the patient safety culture in emergency ward	0.40±3.41	0.42±3.1	0.39±3	0.36±3.08	0.30±3.12	0.46±3.36	0.59±2.67	0.35±3.23	0.51±3.25	0.41±2.65	0.35±3.04	0.40±3.24	0

The findings regarding the analysis of the aspects of the safety culture using multivariate decision-making techniques and TOPSIS indicated that human factors are of the highest priorities among the aspects of the patients’ safety culture.

After forming the decision hierarchy for the priority of patients’ safety culture dimensions, the weights of the criteria were calculated by five experts. The expert’s teams were given the task to formindividual scales by using the given scales. The results obtained from the experts are presented in Table 2.

**Table 2:** Multivariate decision-making matrix of the factors effective on the patients’ safety culture in emergency wards based on the experts’ viewpoints

Criteria	Item	D1	D2	D3	D4	D5
**C1**	Human factors	VG	M	VG	VG	G
	Managerial factors	G	M	P	G	G
	Organizational/environmental factors	P	VP	P	G	G

**C2**	Human factors	VG	G	G	M	VG
	Managerial factors	G	G	M	G	G
	Organizational/environmental factors	G	G	VG	VP	M

**C3**	Human factors	G	G	VG	G	M
	Managerial factors	G	M	G	G	M
	Organizational/environmental factors	G	G	VP	G	P

**C4**	Human factors	VG	VG	G	M	P
	Managerial factors	P	VG	G	M	M
	Organizational/environmental factors	G	M	G	M	P

Managerial, organizational, and environmental factors were the next priorities in a descending order (Table 3).

**Table 3:** The priority of the factors effective on the improvement of the patients’ safety culture in the studied hospitals based on the TOPSIS technique

Rank	Item	Coefficient
**1**	Human factors	0.7995
**2**	Managerial factors	0.3756
**3**	Organizational/environmental factors	0.2005

## 5. Discussion

As the findings regarding the effective human factors on the patients’ safety culture in the emergency wards indicated, the personnel’s perception of the patients’ safety was at a mean level. Moreover, half of the personnel mentioned the problem in the error prevention procedures and systems. Keady and Thaker from the American Medical Institute and the UK Health System argued that the main cause of medical errors is improper function of the systems or shortcomings of the health systems (Keady, 2008; Dalton et al., 2008). Proper designs of health systems can be helpful in preventing the variety of medical errors and help the hospitals in improving the safety levels of the patients.

More than half of the study society believed that, given the nature of tasks in emergency wards, high workload, poor staffing, and more than 40hrs work a week has caused the staff in the emergency wards to work intensively. Dolton et al. (2008) confirmed the same and concluded that working intensively under hard conditions leads to burnouts, increase of human error and lower safety levels (Dalton et al., 2008). Studies have confirmed that one of the reasons of the reduction of patient safety is human error (Pierce, 1999; Firth-Cozens, 2001; Weinger & Gaba, 2014). Analyses of cultural aspects by the experts using multivariate decision-making technique showed that human factor is the most important element in safety improvement programs.

Concerning the managerial factors effective on the improvement of the patients’ safety in the emergency wards, the results showed that the expectations and performance of the wards supervisors and the support from the hospital management were at a mean level. Moreover, 30% of the personnel believed that their supervisor does not pay attention to their recommendations to improve the patients’ safety and 52% of the nurses argued that their supervisor supports them when they perform a task based on the principles of safety. Thus, being supportive and fostering the employees to follow safety codes may result in the promotion of such behaviors. The notable issue concerning the two points was little emphasis on the patients’ safety by different levels of management. According to 40% of the participants, the management of hospital show interest in the patients’ safety only when something goes wrong. In addition, 17% of the personnel believed that improving the patients’ safety is a critical task that has to do with all levels of management. That is, each manager must create a supportive environment in their section such as implementing safety programs and training courses. As a result, the negative consequences of incidents will be minimized. Zaboli et al. emphasized the role of the management of the hospitals in creating a safe environment for the patients and introducing proper safety programs for the employees (Zaboli, 2007). Managers have come to the conclusion that doing preventable errors over and over and imposing threat to the patient’s safety is in contrast with the primary goals of health systems. They also believe that a systemic approach to medical errors not only increases the safety of the patient but also improves the effectiveness of clinical services. It also increases the performance of the management and lowers the costs of services. According to the experts and based on multivariate decision making, managerial factors were the second effective factor and priority in the improvement of patients’ safety culture.

Regarding the organizational and environmental factors, the findings showed that organizational training is reasonably effective on the patients’ safety. About half of the participating nurses believed that the lessons learned from the errors could be used to improve the safety of patients as a learning tool. Apparently, neglecting the lessons learned from the errors is because of the absence of the error reporting system in the health systems. Winer (2008) proved that lack of error reporting system is due to punishing attitudes promoted in health and treatment organizations (Weiner et al., 2008). Kohestani et al. (2007) stated that the fear of the consequences of reporting errors is the main challenge ahead of extending a pharmaceutical error reporting system among students of nursing (Kouhestani & Baghcheghi, 2009). It was recommended to promote a fair and effective culture regarding the patients’ safety field to replace the current culture in the hospitals. The notable point is that a healthy organizational culture does not hesitate to take measures to prevent the repetition of medical errors and preserving patients against future threats that need disciplinary actions.

Teamwork attitudes within and between the wards were at a mean level with a tendency to higher level. About 70% of the personnel believed that there was teamwork attitude in the ward and more than a half of the personnel confirmed good relationships among the staff. On the other hand, 57% of the personnel agreed that there is lack of coordination among the wards. One may conclude that one of the causes of the repetitive errors in the past is the negligence of teamwork attitudes and some sort of individualistic culture. One explanation is that by participating in teamwork, the employees can supervise and support each other, which increases the chance to prevent the repetition of errors and avoids errors before causing problems for the patient. On the other hand, provision of health care services is a teamwork job in nature. Rozovsky and Woods stated that teamwork reduces the probability of personnel errors. Under a teamwork structure, the intensive executive workload and the risk of burnouts are replaced by new responsibilities to create good relationships with other team members (Bellamy, 2005). Marshal et al. (2007) emphasized teamwork and how human factors positively influence preventing errors (Marshall, & Manus, 2007). Concerning open and transparent relationships, 50.9% of the participants confirmed an open environment to share ideas and 43.2% of the personnel argued that they are informed of the errors in their ward. Hustchinson et al. concluded that there was a statistically significant relationship between error reporting rates and the independent safety culture of the patients; this means the increase of the reporting rates positively influences the development of the safety culture in hospitals (Hutchinson, 2009). The results of Bodure showed consistent conclusions (Bodur & Filiz, 2009).

## 6. Conclusion

The findings indicated environmental and organizational factors ranked as the 3^rd^ priority in multivariate decision-making analysis. The human factor, on the other hand, was ranked as the first priority, which must be taken into account in safety improvement programming. Training, cultural works, and the provision of supportive organizational and environmental factors are the most effective steps that can be taken by the management of hospitals.

This study faced a few limitations. Although the patients’ safety culture in public hospitals was prioritized, but it can also be used in other decision-making situations. Also, mathematical models such as TOPSIS can provide accurate estimations.
